# Supplementary material for: Unfavorable Dietary Quality Contributes to Elevated Risk of Ischemic Stroke among Residents in Southwest China: Based on the Chinese Diet Balance Index 2016 (DBI-16)
Source: Nutrients. 2022 Feb 7;14(3):694. doi: 10.3390/nu14030694 (PMC8838893; doi:10.3390/nu14030694)
Supplement: Supplementary file 1 [file nutrients-14-00694-s001.zip › nutrients-1559816-supplementary.pdf]

Table S1 DBI-16 components and standard for scoring[1]

Component	Score	Subgroup	Score	Intake range by energy intake level										
				**1000 kcal**	**1200 kcal**	**1400 kcal**	**1600 kcal**	**1800 kcal**	**2000 kcal**	**2200 kcal**	**2400 kcal**	**2600 kcal**	**2800 kcal**	**3000 kcal**
C1-Cereal	(-12)-12	Cereal	(-12)-12	0g=-12<br>75-95g=0<br>>170g=12	<15g=-12<br>90-110g=0<br>>185g=12	0g=-12<br>125-175g=0<br>>250g=12	<10g=-12<br>175-225g=0<br>>390g=12	<35g=-12<br>200-250g=0<br>>415g=12	<5g=-12<br>225-275g=0<br>>495g=12	<30g=-12<br>250-300g=0<br>>520g=12	0g=-12<br>275-325g=0<br>>600g=12	<50g=-12<br>325-375g=0<br>>650g=12	<75g=-12<br>350-400g=0<br>>675g=12	<100g=-12<br>375-425g=0<br>>700g=12
C2-Vegetable and fruit	(-12)-0	Vegetable	(-6)-0	≥200g=0<br>160-199g=-1<br>Score decreased 1 with intake amount decreased 40g<br>0g=-6	≥250g=0<br>200-249g=-1<br>Score decreased 1 with intake amount decreased 50g<br>0g=-6	≥3000g=0<br>240-299g=-1<br>Score decreased 1 with intake amount decreased 60g<br>0g=-6		≥400g=0<br>320-399g=-1<br>Score decreased 1 with intake amount decreased 80g<br>0g=-6	≥450g=0<br>360-449g=-1<br>Score decreased 1 with intake amount decreased 90g<br>0g=-6		≥500g=0<br>400-499g=-1<br>Score decreased 1 with intake amount decreased 1000g<br>0g=-6			≥600g=0<br>480-599g=-1<br>Score decreased 1 with intake amount decreased 1200g<br>0g=-6
		Fruit	(-6)-0	⩾150g=0; 120-149g=-1<br>Score decreased 1 with intake amount decreased 30g<br>0g=-6			⩾2000g=0; 160-199g=-1<br>Score decreased 1 with intake amount decreased 40g<br>0g=-6		⩾300g=0; 240-299g=-1<br>Score decreased 1 with intake amount decreased 60g<br>0g=-6		⩾350g=0; 280-349g=-1<br>Score decreased 1 with intake amount decreased 70g<br>0g=-6		⩾400g=0; 320-399g=-1<br>Score decreased 1 with intake amount decreased 80g<br>0g=-6	
C3-Milk and dairy products Soybean and soybean products	(-12)-0	Dairy	(-6)-0	≥500g=0<br>Score decreased 1 with intake amount decreased 100g<br>0g=-6		≥350g=0<br>Score decreased 1 with intake amount decreased 70g<br>0g=-6	≥300g=0<br>Score decreased 1 with intake amount decreased 60g<br>0g=-6							
		Soybean	(-6)-0	⩾5g=0	⩾15g=0					⩾25g=0				

				Score decreased 1 with intake amount decreased 1g<br>0g=-6	Score decreased 1 with intake amount decreased 3g<br>0g=-6					Score decreased 1 with intake amount decreased 5g<br>0g=-6		
C4-Animal food	(-12)-8	Red meat and products, Poultry and game	(-4)-4	0g=-3<br>1-5g=-2<br>6-10g=-1<br>11-20g=0<br>21-25g=1<br>26-30g=2<br>31-35g=3<br>>35g=4	0g=-4<br>1-5g=-3<br>6-10g=-2<br>11-15g=-1<br>16-35g=0<br>36-40g=1<br>41-45g=2<br>46-50g=3<br>>50g =4	0g=-4<br>1-10g=-3<br>11-20g=-2<br>21-30g=-1<br>31-50g=0<br>51-60g=1<br>61-70g=2<br>71-80g=-3<br>>80g =4		0g=-4<br>1-15g=-3<br>16-30g=-2<br>31-45g=-1<br>46-55g=0<br>56-70g=1<br>71-85g=2<br>85-100g=3<br>>100g =4		0g=-4<br>1-20g=-3<br>21-40g=-2<br>41-60g=-3<br>61-90g=0<br>91-110g=1<br>111-130g=2<br>131-150g=3<br>>150g=4	0g=-4<br>1-25g=-3<br>26-50g=-2<br>51-75g=-1<br>76-125g=0<br>126-150g=1<br>151-175g=2<br>176-200g=3<br>>200g=4	
		Fish and Shrimp	(-4)-0	0g=-4<br>1-4g=-3<br>5-9g=-2<br>10-14g=-1<br>⩾15g=0	<5g=-4<br>5-9g=-3<br>10-14g=-2<br>15-19g=-1<br>⩾20g=0	<10g=-4<br>10-19g=-3<br>20-29g=-2<br>30-39g=-1<br>⩾40g=0		<5g=-4<br>5-19g=-3<br>20-34g=-2<br>35-49g=-1<br>⩾50g=0		0g=-4<br>1-24g=-3<br>25-49g=-2<br>50~74g=-1<br>⩾75g=0	<25g=-4<br>25-49g=-3<br>50-74g=-2<br>75-99g=-1<br>⩾100g=0	<50g=-4<br>50-74g=-3<br>75-99g=-2<br>100-124g=-1<br>⩾125g=0
		Egg	(-4)-4	0g=-4<br>1-5g=-3<br>6-10g=-2<br>11-15g=-1<br>16-25g=0<br>26-30g=1	<5g=-4<br>6-10g=-3<br>11-15g=-2<br>16-20g=-1<br>21-30g=0<br>31-35g=1		0g=-4<br>1-10g=-3<br>11-20g=-2<br>21-30g=-1<br>31-50g=0<br>51-60g=1		0g=-4<br>1-15g=-3<br>16-30g=-2<br>31-45g=-1<br>46-55g=0<br>56-70g=1			

				31-35g=2<br>36-40g=3<br>>40g=4	36-40g=2<br>41-45g=3<br>>45g =4		61-70g=2<br>71-80g=-3<br>>80g =4	71-85g=2<br>85-100g=3<br>>100g =4		
C5-Empty energy food	0-12	Cooking oil	0-6	≤20g=0<br>21-25g=1<br>>45g=6	≤25g=0<br>26-30g=1<br>>50g=6				≤30g=0<br>31-35g=1<br>>55g=6	≤35g=0<br>36-40g=1<br>>60g=6
		Alcoholic beverage	0-6	Male: ≤ 25g=0; 26-40g=1; score increased 1 with intake amount increased 15g; >100g=6<br>(25g alcohol=750ml beer or 250ml wine or 75g liquor 38°or 50g liquor > 38°)<br>Female: ≤15g=0; 16-25g=1; score increased 1 with intake amount increased 10g; >65g=6<br>(15g alcohol=450ml beer or 150ml wine or 50g liquor 38° or 30g liquor > 38°)						
C6-Condiments	0-12	Addible sugar	0-6	≤25g=0; 26g=1; score increased 1 with intake amount increased 5g; >50g=6						
		Salt	0-6	<2g=0<br>2-3g=1<br>Score increased 1 with intake amount increased 2g<br>>12g=6	<3g=0<br>3-4g=1<br>score increased 1 with intake amount increased 2g<br>>13g=6	<4g=0<br>4-5g=1<br>Score increased 1 with intake amount increased 2g<br>14g=6	<6g=0<br>6-7g=1<br>score increased 1 with intake amount increased 2g<br>>16g=6			
C7-Diet variety	(-12)-0	Diet variety	(-12)-0	≥12 kinds of food (soybean is 5g) =0; score decreased 1 with decreased 1 kinds of food						
C8-Drinking water	(-12)-0	Drinking water	(-12)-0	≥1200ml=0; score decreased 1 with intake amount decreased 100ml; <100ml=-12						

[1]This table has been reproduced from He, Y., et al., Update of the Chinese diet balance index: DBI-16. Acta Nutrimenta Sinica, 2018. 40(06): p. 526-530.
